# Addressing gender inequalities to improve the sexual and reproductive health and wellbeing of women living with HIV

**DOI:** 10.7448/IAS.18.6.20302

**Published:** 2015-12-01

**Authors:** Avni Amin

**Affiliations:** Department of Reproductive Health and Research, World Health Organization, Geneva, Switzerland

**Keywords:** gender inequalities, stigma, discrimination, laws, sexual and reproductive health

## Abstract

**Introduction:**

Globally, women constitute 50% of all persons living with HIV. Gender inequalities are a key driver of women's vulnerabilities to HIV. This paper looks at how these structural factors shape specific behaviours and outcomes related to the sexual and reproductive health of women living with HIV.

**Discussion:**

There are several pathways by which gender inequalities shape the sexual and reproductive health and wellbeing of women living with HIV. First, gender norms that privilege men's control over women and violence against women inhibit women's ability to practice safer sex, make reproductive decisions based on their own fertility preferences and disclose their HIV status. Second, women's lack of property and inheritance rights and limited access to formal employment makes them disproportionately vulnerable to food insecurity and its consequences. This includes compromising their adherence to antiretroviral therapy and increasing their vulnerability to transactional sex. Third, with respect to stigma and discrimination, women are more likely to be blamed for bringing HIV into the family, as they are often tested before men. In several settings, healthcare providers violate the reproductive rights of women living with HIV in relation to family planning and in denying them care. Lastly, a number of countries have laws that criminalize HIV transmission, which specifically impact women living with HIV who may be reluctant to disclose because of fears of violence and other negative consequences.

**Conclusions:**

Addressing gender inequalities is central to improving the sexual and reproductive health outcomes and more broadly the wellbeing of women living with HIV. Programmes that go beyond a narrow biomedical/clinical approach and address the social and structural context of women's lives can also maximize the benefits of HIV prevention, treatment, care and support.

## Introduction

Globally, women constitute half of all persons living with HIV. In sub-Saharan Africa, the region with the highest burden of HIV, women constitute 57% of persons living with HIV; and adolescent girls and young women are twice as likely to be living with HIV as compared to boys and young men. In low- and middle-income countries, female sex workers are 13.5 times more likely to be living with HIV as compared to the general population of women in reproductive age groups [1]. Globally, transgender women are 49 times more likely to be living with HIV as compared to all adults of reproductive age groups [2–4].

The sexual and reproductive health needs of women living with HIV require particular attention because these women are disproportionately vulnerable to certain reproductive health problems as compared to HIV-negative women and also in relation to the prevention of vertical transmission of HIV. Studies show that, as with women who are HIV negative, women living with HIV have high rates of unintended pregnancy and low rates of contraceptive use including condom use [5–9]. In sub-Saharan Africa, women living with HIV are significantly more likely to die during pregnancy or the postpartum period as compared to HIV-negative women [10,11]. Globally, women living with HIV are also more likely to have a higher incidence and progression of cervical neoplasia as compared to women who are HIV negative [12].

There has been increasing attention given to certain aspects of reproductive health of women living with HIV, particularly in the context of preventing vertical transmission of HIV. However, much of this focus has largely addressed the biomedical/clinical and health systems factors [13–15]. There has been less attention to a more holistic response that goes beyond disease prevention and addresses the sexual, emotional and mental health as well as social and economic wellbeing of women living with HIV as a legitimate focus of programming and research in its own right [16,17]. This state of affairs stands in stark contrast to what women living with HIV have articulated as their needs and priorities. These needs include the importance of addressing gender inequalities, violence against women, financial security and social support, reproductive health beyond pregnancy, and sexuality in a positive framework [18]. The UNAIDS Gap Report [3] highlights women living with HIV as one of the 12 priority populations. The report identifies stigma and discrimination, gender inequalities, and punitive laws and policies as three of the top four reasons for their vulnerability.

Nearly two decades of research and programming have highlighted that gender inequalities are a key structural driver of women's vulnerability to acquiring HIV. The importance of addressing gender inequalities is well recognized in key global commitments to ending HIV. Some countries are beginning to address them as part of their national HIV and AIDS responses [19,20]. However, concrete actions on a significant scale and in a sustained manner with concomitant resources are yet to materialize. The pathways by which gender inequalities shape women's risk of acquiring HIV are increasingly being mapped out, particularly as they relate to the intersections of intimate partner violence and HIV [21–23]. There is a small, but increasing body of evidence on interventions that work to address gender inequalities as a structural driver of women's risk of becoming infected with HIV, such as those that promote egalitarian gender norms, empower women and girls economically and in their sexual and reproductive decision-making, and reduce violence against women [23–27].

While gender inequalities affect HIV-negative women as well as women living with HIV in many similar ways, the latter face unique challenges related to stigma and discrimination, as well as pressures related to their sexual and childbearing decisions, economic security, mental health and emotional wellbeing. This paper describes how gender inequalities shape the sexual and reproductive health and wellbeing of women living with HIV, specifically via the following pathways: (1) unequal power relations, harmful gender norms and violence against women; (2) women's unequal access to and control over economic resources; (3) stigma and discrimination; and (4) punitive laws and gender-discriminatory policies. These pathways are examined in terms of four interrelated outcomes: (1) disclosure of HIV status; (2) ability to have safe and pleasurable sex; (3) fulfilment of fertility intentions and enabling of reproductive choices; and (4) management of treatment. The concept of wellbeing is included to underscore the importance of considering mental and emotional health as well as social and economic factors.

## Unequal power relations in sexual and reproductive decision-making: the role of harmful gender norms and violence against women

In many settings, gender norms privilege men's control over women or perpetuate unequal power relations. These norms prevent women from having autonomy in sexual and reproductive health decisions. Surveys of women of reproductive age (e.g. demographic and health surveys) show that in many settings, a large proportion of married women, especially young women, do not have a final say in their own healthcare decisions [3,28,29]. Analysis of sexual behaviours of women and men from surveys shows that in general, married women find negotiation of safer sex and condom use much more difficult than do single women [30].

In many societies, women living with HIV, like others, face tremendous social pressures to bear children. Women gain status and their worth is proven through their fertility. Research highlights the importance of partners’ dominance in decision-making with respect to condom use and desire for children in shaping the sexual and reproductive decisions of women living with HIV as well as in the uptake of prevention of mother-to-child HIV transmission (PMTCT) services [31–34]. Hence, women, including those living with HIV, face pressures to have unprotected sex in order to conceive or are unable to use contraception because of such social norms [18,35–37].

Gender norms related to sexuality confer different expectations for women and men to have consensual sex [38–40]. For women, a central issue is that of freedom from violence, which is a stark expression of men's power, control and entitlement over women. Globally 30% of women have experienced physical and/or sexual violence by an intimate partner in their lifetime [41,42]. Data show that intimate partner violence against women is associated with a 1.5-fold increase in risk of sexually transmitted infections (STIs) or HIV in some regions [41]. Data on prevalence of intimate partner violence among women living with HIV are not easily obtained. However, one systematic review of studies from the United States of America highlighted a higher proportion of women living with HIV experiencing partner violence as compared to women in the general population [43]. A large body of studies from sub-Saharan Africa show that women's fear or experience of violence are a major barrier to HIV disclosure [44,45]. Studies on HIV disclosure outcomes among women living with HIV show that rates of negative outcomes, including violence, range from 3 to 15% and up to 59% in a couple of studies [46–48]. Studies also show an association between partner violence and lower uptake of PMTCT, continued or increased sexual risk behaviours and poor adherence to antiretroviral therapy – in part explained by stress, poor mental health, and a lack of control over health-promoting behaviours [43,49–53].

## Unequal access to and control over economic resources: the role of food insecurity and lack of property and inheritance rights

An increasing number of studies highlight that, while antiretroviral therapy (ART) access has improved, there continue to be socio-economic barriers to uptake of and adherence to treatment. Food insecurity has been identified as a key barrier to ART adherence and quality of life for people living with HIV by a number of studies [54–56]. Women are disproportionately susceptible to food insecurity because of their lack of access to and control over economic resources in the form of ownership of land, assets and other property, and their lower access to formal employment than men. Research from sub-Saharan Africa and South Asia highlights how women living with HIV are denied their property and inheritance rights by relatives when their husbands die due to HIV-related conditions [57–60].

This denial of land and property rights contributes to food insecurity, which in turn increases sexual risk taking (e.g. transactional or commercial sex) and limits women's ability to leave abusive relationships. For example, a study from Swaziland and Botswana highlighted that food insecurity among women was associated with significantly higher odds of inconsistent condom use with a non-primary partner, transactional sex and lack of control in sexual relationships, but that these associations were weaker among men [61]. Similar findings were shown in a qualitative study on food insecurity among women living with HIV in Uganda [62]. Studies also highlight women's economic dependency and their fear of being abandoned as a barrier to HIV disclosure [44,45,63,64]. Other adverse consequences of food insecurity on women living with HIV are in relation to their increased nutritional and energy requirements during pregnancy and lactation as well as the increased stress and burden on them to procure food and clean water for family members, including children who may also be living with HIV [65–67].

## Stigma and discrimination

Stigma and discrimination are among key barriers that women living with HIV face in achieving their sexual and reproductive health. While all those who are living with HIV can face stigma because of judgments made about their behaviours by families and communities, women are more likely to be blamed because many societies have different expectations and standards for women's sexual conduct than for men's [68,69]. Moreover, in sub-Saharan Africa, as women are more likely to be tested first in the context of PMTCT programmes, they are also more likely to be blamed for bringing HIV into the family [44,45,70]. This potential consequence is likely not only to affect women's willingness to disclose their HIV status, but also to compromise their safety due to threats or experience of violence. Some women living with HIV report rejection of sexual relations by their partners or inability to find sexual partners because of their HIV status [18,71]. Women living with HIV may also experience internalized stigma that includes fear and anxiety that partners may not find them attractive [70,72,73]. In some settings, HIV programme staff discourage women living with HIV to have sex or blame them as being irresponsible if they have unprotected sex, which can affect their sexual, emotional and mental health and wellbeing [74,75].

For a number of women living with HIV who want children, there are pressures from institutions such as healthcare to not bear children [76]. Data from Bangladesh, the Dominican Republic and Ethiopia show that between a quarter to nearly half of all women living with HIV were advised by health workers to not have children [77]. Reports of women living with HIV being coerced into sterilizations have occurred in several settings (e.g. Bangladesh, Chile, Dominican Republic, Honduras, El Salvadaor, Mexico, Nicaragua and Namibia) [3,78]. Several countries surveyed as part of the stigma index (i.e. a survey-based tool to assess or measure levels of stigma experienced by people living with HIV) reported the proportion of women living with HIV who were denied family planning services in the last 12 months to be at least 10% [4]. These data highlight the contradictory pressures that women living with HIV face in relation to their fertility intentions and reproductive choices. The enactment of these contradictory pressures on women by healthcare institutions violates their reproductive rights.

## Laws that criminalize HIV transmission and gender-discriminatory HIV policy responses

Laws that criminalize HIV transmission, exposure and non-disclosure are not only unjust and difficult to enforce, but make for poor public health practice and outcomes by disempowering those living with HIV and discouraging them from testing, accessing treatment programmes or disclosing their HIV status [79]. Despite this, 61 countries have adopted laws that criminalize HIV transmission, while prosecutions for non-disclosure, exposure and transmissions have been recorded in at least 49 countries [3]. These laws are being adopted in a context of rapid expansion of HIV testing of pregnant women through PMTCT programmes. In West and Central Africa, laws criminalize women who transmit HIV to the foetus or child. This puts women in an impossible quandary, given that many are unable to demand condom use or disclose their HIV status due to fears of violence or abandonment by their partners [79]. Data show that punitive laws and law enforcement practices related to sex work and injecting drug use also contribute to stigma, violence and other rights violations against women living with HIV from key populations [3,79].

HIV policies have often failed to take into account gender inequalities in ways that further contribute to discrimination against women. Such policies have also failed to address the reasons behind men's lower access to HIV services. For example, HIV testing and counselling and disclosure has a distinct gendered pattern and dimension [44,45,80]. In a number of countries, more women are tested and know their HIV status compared to men, particularly in the context of women's higher frequency of use of maternal and child health services [81]. Studies from sub-Saharan Africa show that masculine norms and stigma prevent men from seeking HIV testing services [82,83]. Men use their partners' HIV status as a proxy for their own [45]. At the same time, an increasing number of countries are putting in place partner notification policies [45]. Hence, the onus of disclosure is on women, even as it brings with it the risk of violence and other negative consequences.

A number of countries with the highest burden of HIV among women and children have started to implement lifelong ART (Option B +) for all pregnant and postpartum women living with HIV and their infants [81]. The public health rationale and benefits for implementing Option B+ have been well established [84]. However, there is less consideration of the implications of early initiation and lifelong treatment, regardless of CD4+ count, on women that takes into account the gendered realities of their daily lives [85]. Data from Malawi, South Africa and Tanzania suggest that, while women are motivated to initiate and adhere to ART during pregnancy and post-partum periods in order to prevent HIV transmission to their child, they are less motivated to continue thereafter [86–88]. Qualitative data from Malawi, Tanzania and Uganda suggest that women living with HIV appreciate the positive benefits of Option B+, including the ability to prevent transmission to their children and partners, their own improved health and reduced stigma. However, they raise concerns about treatment and adherence in relation to the following: the lack of food security and nutrition that is required to maintain treatment; the requirement to disclose their HIV status, especially for those who face or fear partner violence; lack of information, support and counselling; and the side effects of treatment [87,89].

## Conclusions

**Figure 1:**
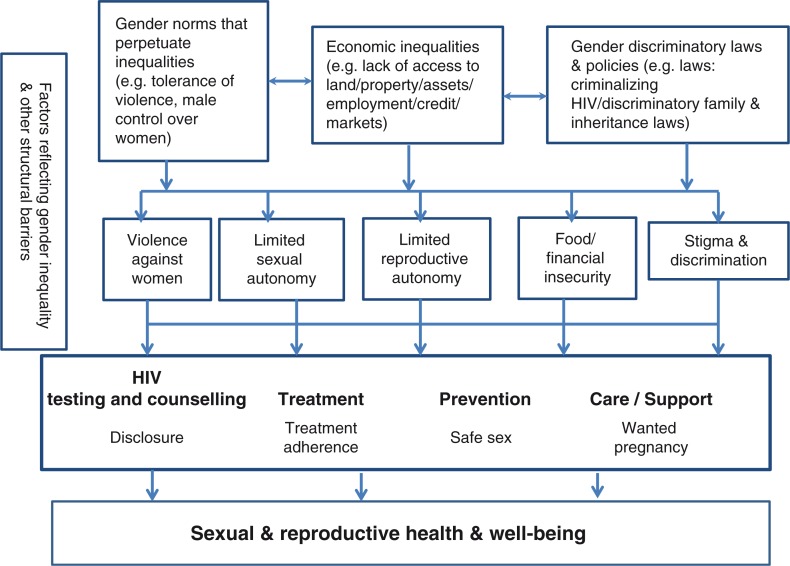
Pathways explaining how gender inequalities shape the sexual and reproductive health and wellbeing of women living with HIV.

Addressing gender inequalities is central to improving the sexual and reproductive health outcomes and more broadly, the wellbeing of women living with HIV. Even as HIV prevention, treatment and care services for women living with HIV are being expanded and bringing many benefits, the context of gender inequalities is undermining these efforts. Figure 1 summarizes the pathways by which gender inequalities shape the sexual and reproductive health and wellbeing of women living with HIV.

This paper highlights the importance of interventions for women living with HIV to promote egalitarian and non-violent norms along with equitable decision-making between women and men. It also highlights the importance of interventions to address economic inequalities that contribute to food insecurity, such as interventions that promote land, property and inheritance rights of women living with HIV. Stigma and discrimination, particularly in healthcare settings, needs to be addressed in order to support the reproductive choices of women living with HIV. Strong advocacy is needed to repeal laws that criminalize HIV transmission. Finally, it is not enough to design HIV policies from a narrow biomedical/clinical and health systems framework. Instead, policies must take into account the social and structural context of women's lives from the very inception, so that women living with HIV feel less isolated and are more empowered to make informed choices and decisions with respect to their health and wellbeing. The evidence for effective women-centred approaches is limited. One such example is a study to improve the sexual and reproductive health of Canadian women living with HIV. As part of this study, a framework was developed to identify the elements of a women-centred model of care that addresses their physical health needs (i.e. from a clinical and biomedical perspective) as well as their social, emotional, mental, spiritual and cultural needs more broadly. The framework considers gender along with other intersecting social inequalities. It highlights the needs of women living with HIV for safety, respect, acceptance, self-determination, access to social and other supportive services, tailored and culturally sensitive information, and peer support, among others [90]. While this model is being empirically tested in one setting, it needs to be further applied in low- and middle-income country settings. A more holistic social science research agenda is needed to provide women-centred services to women living with HIV and promote their sexual and reproductive health and wellbeing – one that is grounded in social justice and human rights.

## Author information

Avni Amin is a technical officer for violence against women at the WHO. She has worked on issues of gender equality, violence against women and their linkages to sexual and reproductive health and HIV for the last 18 years.
